# Microvesicles Derived from Mesenchymal Stem Cells Enhance Survival in a Lethal Model of Acute Kidney Injury

**DOI:** 10.1371/journal.pone.0033115

**Published:** 2012-03-14

**Authors:** Stefania Bruno, Cristina Grange, Federica Collino, Maria Chiara Deregibus, Vincenzo Cantaluppi, Luigi Biancone, Ciro Tetta, Giovanni Camussi

**Affiliations:** 1 Department of Internal Medicine and Molecular Biotechnology Center, University of Torino, Torino, Italy; 2 Fresenius Medical Care, Bad Homburg, Germany; Universidade de Sao Paulo, Brazil

## Abstract

Several studies demonstrated that treatment with mesenchymal stem cells (MSCs) reduces cisplatin mortality in mice. Microvesicles (MVs) released from MSCs were previously shown to favor renal repair in non lethal toxic and ischemic acute renal injury (AKI). In the present study we investigated the effects of MSC-derived MVs in SCID mice survival in lethal cisplatin-induced AKI. Moreover, we evaluated *in vitro* the effect of MVs on cisplatin-induced apoptosis of human renal tubular epithelial cells and the molecular mechanisms involved. Two different regimens of MV injection were used. The single administration of MVs ameliorated renal function and morphology, and improved survival but did not prevent chronic tubular injury and persistent increase in BUN and creatinine. Multiple injections of MVs further decreased mortality and at day 21 surviving mice showed normal histology and renal function. The mechanism of protection was mainly ascribed to an anti-apoptotic effect of MVs. *In vitro* studies demonstrated that MVs up-regulated in cisplatin-treated human tubular epithelial cells anti-apoptotic genes, such as Bcl-xL, Bcl2 and BIRC8 and down-regulated genes that have a central role in the execution-phase of cell apoptosis such as Casp1, Casp8 and LTA. In conclusion, MVs released from MSCs were found to exert a pro-survival effect on renal cells *in vitro* and *in vivo*, suggesting that MVs may contribute to renal protection conferred by MSCs.

## Introduction

Several studies demonstrated that the administration of *in vitro* expanded bone marrow mesenchymal stem cells (MSCs) improves acute kidney injury (AKI) [1]; in particular the infusion of MSCs was shown to favor functional and morphological recovery in rodent models of AKI induced by cisplatin [2]–[4], glycerol [5] and ischemia-reperfusion injury [6], [7]. However, the mechanisms involved in renal regeneration induced by MSCs remain controversial. Despite the reports that MSCs may localize within the regenerating tubules [2], [5], only a transient accumulation of MSCs in the renal vasculature seems to be required for renal repair [7]. MSCs favor tubular regeneration, which is mainly sustained by the division of tubular cells survived to injury [8] by a paracrine mechanism. Moreover, the study of Bi et al. [4] showed that the factors produced by the cells may replace the therapeutic effect of MSCs.

Besides soluble factors, we demonstrated that microvesicles (MVs) derived from adult human MSCs contribute to kidney repair in glycerol- [9] and ischemia-reperfusion [10] -induced AKI. MVs are small vesicles released by cells that carry membrane and cytoplasmic constituents of the cells from which they originate [11]–[14]. Ratajczak et al., demonstrated that embryonic stem cell derived MVs may reprogram hematopoietic progenitors by horizontal transfer of mRNA and protein delivery [15]. Subsequent studies showed that beside embryonic, also adult stem cell-derived MVs shuttle selected patterns of mRNA and miRNA, suggesting a role of MVs in the genetic exchange between cells [15]–[18]. Quesemberry et al. [19] proposed that MVs play a critical role in the continuum model of stem cell biology.

We recently characterized the surface receptors and the mRNA/miRNA content of MVs derived from human MSCs [9], [18]. MSC-derived MVs were shown to express several adhesion molecules some of which, namely CD44 and CD29, were found to be instrumental in MV internalization in renal tubular cells [9]. The mRNAs and miRNAs content of MVs derived from MSCs associated with the mesenchymal differentiative phenotype and with several cell functions involved in the control of transcription, proliferation and cell immune regulation [9], [18]. We also demonstrated the MV-mediated transfer of functional mRNAs and miRNAs to tubular epithelial cells [9], [18], [20].

In this work, we focused on the effects of MSC-derived MVs on survival in a lethal model of AKI induced by cisplatin in SCID mice. Moreover, we evaluated *in vitro* the effect of MVs on cisplatin-induced apoptosis of renal tubular epithelial cells and the molecular mechanisms involved.

## Results

### MVs derived from MSCs reduced mortality induced by cisplatin

**Figure 1:**
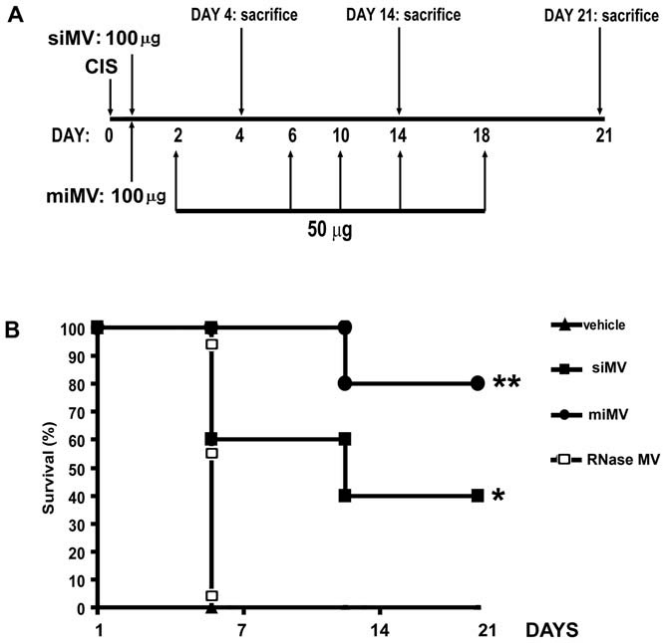
Schematic representation of the protocol of cisplatin induced AKI and MV administration regimens and survival curves. A) Graph showing time-points of cisplatin administration, siMVs or miMVs and the time-points of sacrifice. B) Survival curves of SCID mice with cisplatin induced AKI treated with different regiments of MVs administration. All mice receiving vehicle alone died within 5 days. Mice that received siMVs or miMVs injections survived significantly longer than control mice treated with vehicle alone or with a si(RNase-inactivated)MVs. Data was analysed via a log-rank test: * *p*<0.05 siMV *vs* CIS; ** *p*<0.05 miMV *vs* siMV. Abbreviations: vehicle = cisplatin treated mice injected with vehicle alone; siMV = cisplatin treated mice with single injection of MVs; miMV = cisplatin treated mice with multiple injection of MVs; RNase MV = cisplatin treated mice injected with a single dose of MVs pre-treated with RNase.

SCID mice are known to be very sensitive to cisplatin treatment [3]. In our experimental setting, SCID mice invariably died within 5 days. Survival curves of SCID mice with AKI given vehicle alone or MVs are shown in figure 1 and table 1. Two regimens of MV administration were used. The single injection (siMVs) of 100 µg MVs 8 hours after cisplatin administration significantly increased survival to 60% at day 14 and 40% at day 21, in respect to mice treated with vehicle alone or with a single dose of RNase-inactivated MVs. Multiple injections of MVs (miMVs) (Figure 1) further increased survival to 80% at day 21.

**Table 1:** Body weight, survival, renal function and morphology in SCID mice injected with cisplatin and different regimens of MVs.

		DAY 4				DAY 14				DAY 21			
	Control	CIS	CIS+siMV	CIS+RNase-MV	CIS+miMV	CIS	CIS+siMV	CIS+RNase-MV	CIS+miMV	CIS	CIS+siMV	CIS+RNase-MV	CIS+miMV
**Body weght** (g)	24.3±1.6	16.8±1.4	19±1.8	17.6±1.4	18.9±1.3	-	15.5±2.1	-	20.8±0.17^†^	-	14,7±1,9	-	23,1±3,3^†^
**BUN** (mg/dl)	24±4	123±12.6	75±10.9^*^	109±9.7	38±2.15^*^,^†^	-	71±9.4	-	26±6^†^	-	69±23	-	23.3 ±1.2^†^
**Crea** (mg/dl)	0.2±0.01	0.8±0.2	0.56±0.15*	0.8±0.35	0.4±0.05^*^,^†^	-	0.42±0.15	-	0.36±0.07^†^	-	0.40±0.16	-	0.28±0.03^†^
**% of survival**	100	100	100	100	100	0	60	0	80	0	40	0	80
**Casts** (n/HPF**)**	0	3.7±1.56	0.26±0.13^*^	2.9±1.65	0.1±0.04^*^,^†^	-	0.3±0.3	-	0^†^	-	0.55±0.23	-	0^†^
**Tubular Necrosis** (n/HPF)	0	3.7±1.03	1.8±0.32^*^	3.4±0.9	0^*^, ^†^	-	0.75±0.25	-	0^†^	-	2.25±0.25	-	0^†^

Results are expressed as mean±SD; ANOVA with Dunnet’s multicomparison test:

* *p<*0.05 siMV and miMV treatments *vs* cisplatin (CIS);

† *p<*0.05 miMV *vs* siMV.

CIS = cisplatin injection; CIS+siMV = cisplatin treated with single injection of MVs; CIS+RNase-MV = cisplatin treated with injection of MV pre-treated with RNase; CIS+miMV = cisplatin treated with multiple injection of MVs.

### MSC-derived MVs improved renal function and morphology in cisplatin-induced AKI

Blood urea nitrogen (BUN) and creatinine levels peaked at day 4 and stabilized to high values until death in mice treated with cisplatin and vehicle alone or with a single dose of RNase-inactivated MVs. As shown in table 1, the siMVs improved renal function at day 4 but in AKI mice surviving at days 14 and 21, BUN and creatinine levels remained more elevated than in cisplatin-untreated controls. In mice receiving miMVs, BUN and creatinine levels significantly decreased in parallel with improved survival, to reach levels not statistically different from those of cisplatin-untreated controls (Table 1).

**Figure 2:**
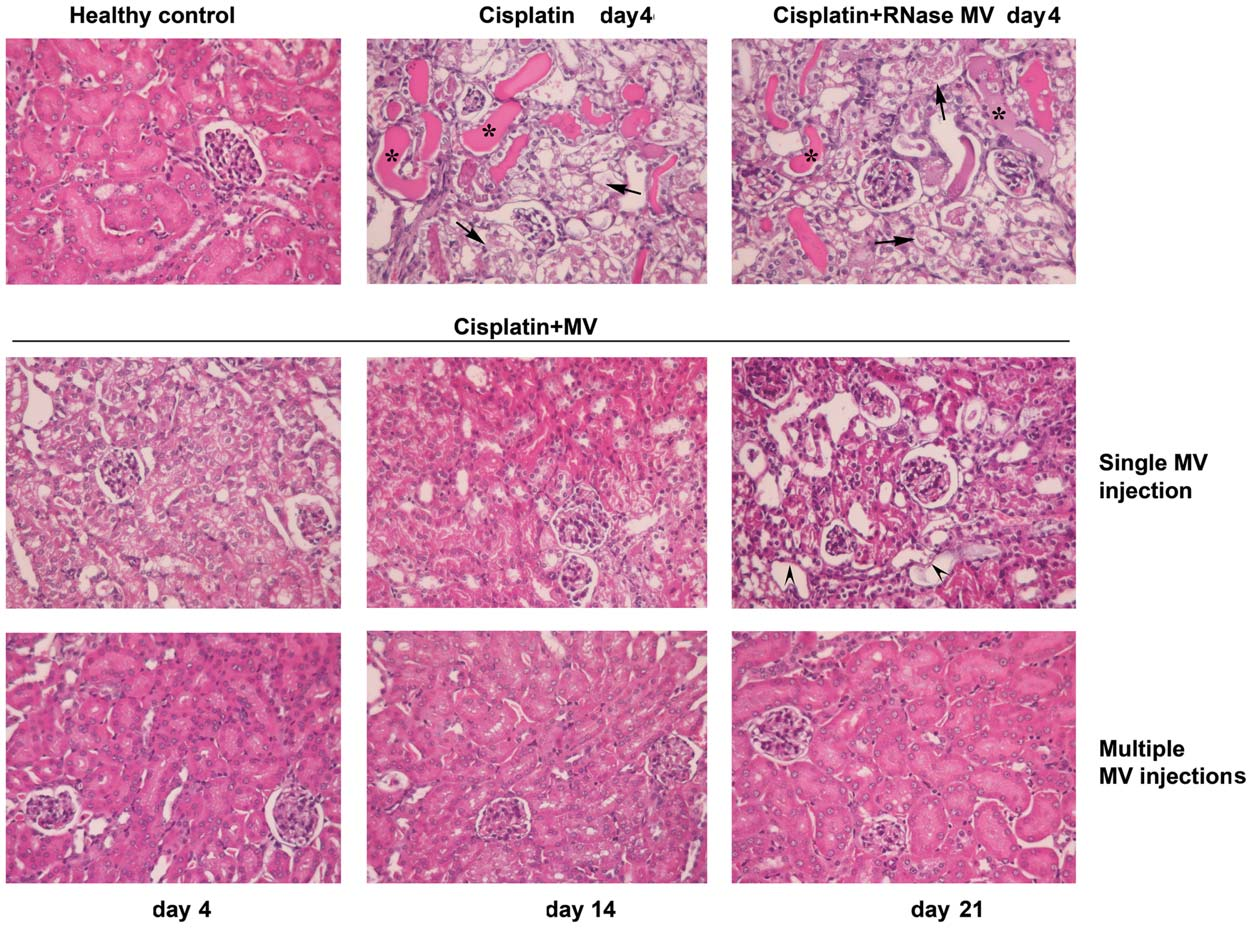
MV infusion protects SCID mice with cisplatin-induced AKI from tubular injury. Representative micrographs of renal histology of healthy SCID mice and of SCID mice treated with cisplatin and injected with vehicle alone or with MV pre-treated with RNase or with different regiments of MVs (single or multiple injections) and sacrificed at different time points (day 4, 14 and 21). Original Magnification: ×200. The typical aspect of intra-tubular casts, tubular necrosis and tubular atrophy are respectively shown by asterisks, arrows and head arrows.

At day 4 after cisplatin injection, kidneys of mice treated with vehicle alone or with a single injection of RNase-inactivated MVs showed severe tubular lesions (Figure 2 and Table 1), consisting in loss of brush border, flattening and loss of the tubular epithelium, nuclear fragmentation, luminal cell debris and hyaline casts. No histological glomerular changes were detectable.

Treatment with a siMVs attenuated tubular injury on days 4 (Figure 2 and Table 1). In particular, protein tubular casts were absent and the extent of necrosis was reduced in respect to mice given vehicle alone. At day 14, a further improvement in tubular injury was observed in survived mice treated with a siMVs. However, a chronic tubular injury persisted up to day 21. At this time several tubules were atrophic. No protein casts were observed at this time.

Treatment with miMVs was significantly more effective than siMVs. At day 21, the renal structure appeared normal (Figure 2 and Table 1).

**Figure 3:**
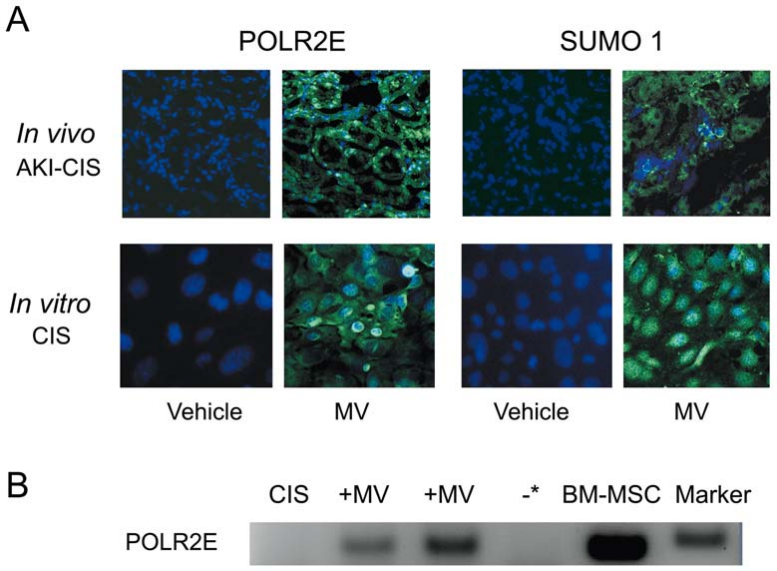
*De novo in vitro* and *in vivo* expression of human proteins after cisplatin and MVs treatment. A) Representative confocal micrographs showing the nuclear and cytoplasmatic expression of human POLR2E and SUMO-1 proteins *in vivo,* in kidney sections of cisplatin-AKI (AKI-CIS) mice treated or not with MVs and sacrificed 48 hours later, and *in vitro* by TECs treated with cisplatin and cultured in the absence (vehicle) or in the presence of 50 µg of MVs (MV) for 24 hours. Nuclei were counterstained with Hoechst dye. Original magnification: ×400 for kidney sections and ×630 for TECs. B) 1×10^5^ TECs treated with cisplatin and cultured in the absence (CIS) or in the presence of two different preparations of MVs (+MV) for 1 hour were analysed by RT-PCR for specific human mRNA *POLR2E*. Bands of PCR products specific for human *POLR2E* of the expected size (90 pb) were detected in a 4% agarose gel electrophoresis. As positive control the extract of human bone marrow-derived MSCs (BM-MSC) was used. The * indicates the control without cDNA.

To investigate the possibility that specific human mRNA shuttled by MVs could be translated in proteins in murine tubular cells after cisplatin induced AKI, we used as reporters *SUMO-1* and *POLR2E*, which mRNAs are present in MVs derived from human MSCs and that are transferred from MVs to renal cells [9]. Using anti human POLR2E and SUMO-1 antibodies, *de novo* expression of human proteins with a nuclear and cytoplasmatic localization could be detected in tubules of mice with cisplatin-AKI treated with MVs but not in those untreated, indicating that specific mRNA shuttled by MVs can be translated into proteins *in vivo* (Figure 3 *)*.

**Figure 4:**
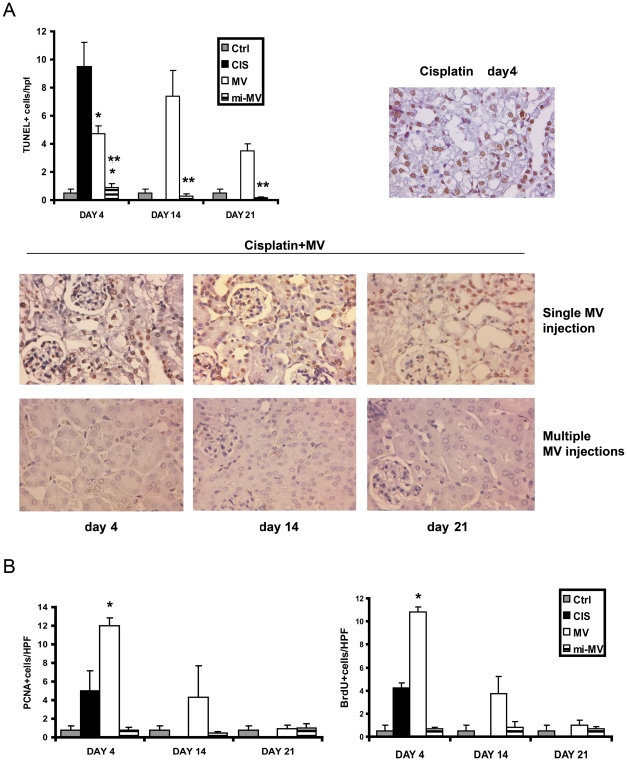
Renal cell apoptosis and proliferation in cisplatin-AKI mice untreated or treated with different regiments of MVs. A) Quantification of Tunel-positive cells/high power field (hpf) at different time points. Data are expressed as mean ±SD of 8 different mice for each experimental condition. ANOVA with Dunnet’s multicomparison test was performed: * *p*<0.05 siMVs or miMVs *vs* CIS; ** *p*<0.05 miMVs *vs* siMVs. Representative micrographs of Tunel staining of renal sections of cisplatin mice given vehicle alone (day 4) and of cisplatin mice treated with different regiments of MVs at different time points (4, 14 and 21 days). Original magnification: ×400. B) Quantification of PCNA positive cells/hpf and of BrdU positive cells/hpf at different time points. BrdU was injected intraperitoneally for 2 successive days before mice being killed. Data are expressed as mean ±SD of 8 different mice for each experimental condition. ANOVA with Dunnet’s multicomparison test was performed: * *p*<0.05 siMVs versus CIS. Abbreviations: Ctrl = healthy mice; CIS = cisplatin treated mice injected with vehicle alone; MV = cisplatin treated mice with single injection of MVs.

Since renal tubular apoptosis was suggested as a mechanism of cisplatin induced AKI [21], [22], we investigated whether MVs exert an anti-apoptotic activity on tubular cells of AKI mice. At day 4 after cisplatin administration, numerous Tunel-positive cells were detected in renal section of cisplatin-mice given vehicle alone (Figure 4). Administration of a siMVs, significantly reduced renal apoptotic cells at day 4, but they remained elevated at day 14 and 21 (Figure 4). In contrast, in mice treated with miMVs, a sustained significant decrease of apoptosis was observed and at day 21 apoptosis was almost absent.

The effect of MV-treatment on tubular cell regeneration in SCID mice with AKI was explored by evaluating PCNA expression and by BrdU uptake. In mice treated with the single dose, a significant increase in tubular cell proliferation was observed as an attempt to counteract the tubular cell loss due to apoptosis (Figure 4). In mice treated with multiple dose, where apoptosis was minimal or absent, no significant increase in tubular cell proliferation was observed (Figure 4). 

### Effect of MVs on apoptosis and apoptotic gene expression by cultured human tubular epithelial cells treated with cisplatin

**Figure 5:**
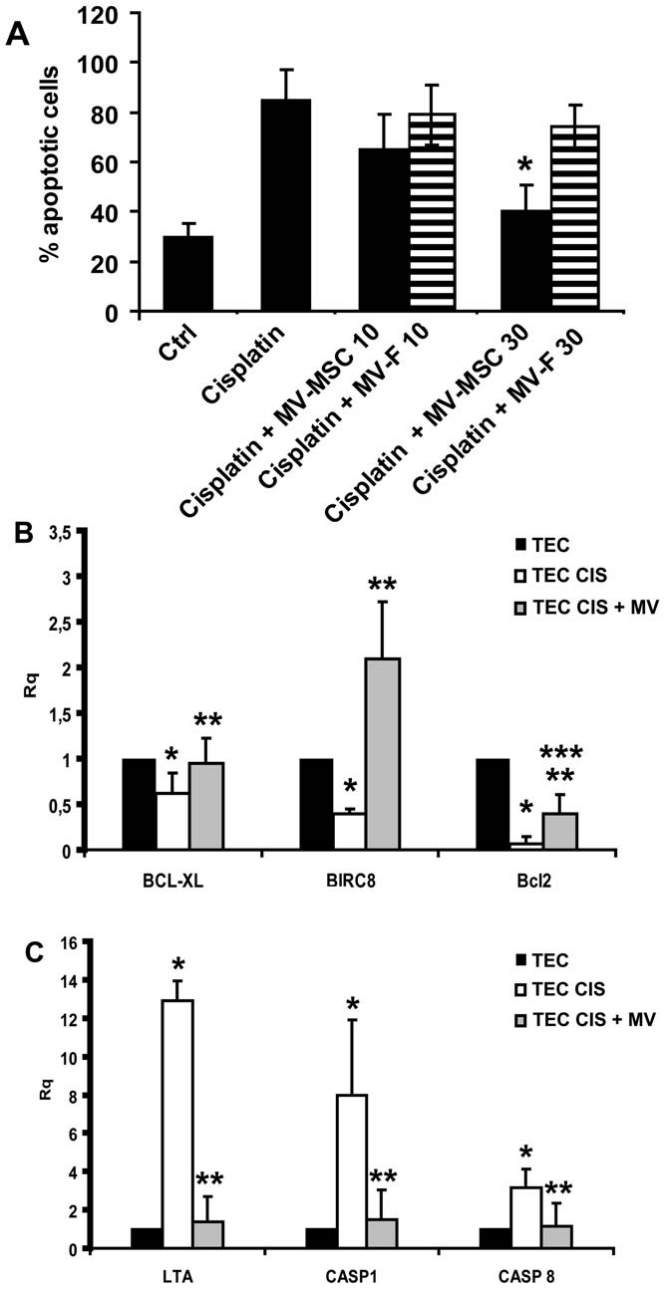
*In vitro* anti-apoptotic effects of MVs on TECs. A) The percentage of apoptotic TECs after incubation with 5 µg/ml of cisplatin was evaluated by the Tunel assay. TECs were incubated in the presence of cisplatin with or without different doses of MVs derived from BM-MSCs or fibroblasts (10 or 30 µg/ml) and 3% FCS (Ctrl = TECs incubated 48 hours in the presence of 3% FCS only). Results are expressed as mean±SD of 4 different experiments. Analyses of variance with Newmann-Keuls multicomparison test was performed: *p<0.05 MVs (30 µg) *vs* vehicle alone. B) Histograms showing the relative expression (Rq) of different anti-apoptotic genes in cisplatin (TEC CIS) and cisplatin-MV treated tubular cells (TEC CIS+MV) in respect to control cells treated with vehicle alone (TEC). Experiments are performed in triplicate. Data was analysed via a Student’s *t* test (unpaired, 2-tailed); * *p<*0.05 TEC CIS vs TEC; ** *p*<0.05 TEC CIS+MV vs TEC CIS; *** *p*<0.05 TEC CIS+MV vs TEC. C) Histograms showing the relative expression (Rq) of pro-apoptotic genes in cisplatin (TEC CIS) and cisplatin-MV treated tubular cells (TEC CIS+MV) in respect to control cells (TEC). Experiments are performed in triplicate. Data was analysed via a Student’s *t* test (unpaired, 2-tailed); * *p<*0.05 TEC CIS vs TEC; ** *p*<0.05 TEC CIS+MV vs TEC CIS.

reatment of human tubular epithelial cells (TECs) with cisplatin *in vitro* induced apoptosis which was significantly inhibited by different doses of MSC-derived MVs (Figure 5). Instead, MVs derived from human fibroblasts did not significantly inhibit TEC apoptosis (Figure 5A). 

To investigate the mechanism involved in the anti-apoptotic effect of MVs we compared the gene expression profile of untreated TECs with TECs treated with cisplatin, in the presence or absence of MVs. After 24 hours of stimulation with cisplatin the activation of programmed cell death pathways in TECs was observed, as detected by the activation of genes involved in growth arrest conditions, such as *GADD45A*, and in apoptosis such as *Bcl-10, CASP-1, CASP-8, LTA, TP73* and *CASP-10*. Moreover, the down-regulation of anti-apoptotic genes such as *Bcl2, Bcl-XL, Akt1* and *TRAF2* was present (not shown). Comparing the cisplatin-treated TECs exposed or not to MVs, up-regulation of some genes involved in the inhibition of the apoptosis and the down-regulation of genes involved in the execution-phase of apoptosis could be observed (Table 2). 

**Table 2:** List and function of genes that significantly differ between cisplatin-treated human tubular cells stimulated or not with MVs.

TEC CIS +MV versus TEC CIS	Official Symbol	Functions of encoded protein
**Up-regulated**	*BCL2*	integral outer mitochondrial membrane protein that blocks the apoptotic death
	*BCL2L1(BCL-XL)*	protein belongs to the BCL-2 protein family: the longer isoform acts as an apoptotic inhibitor and the shorter form acts as an apoptotic activator
	*BIRC8*	a testis-specific inhibitor of apoptosis, but is role is not completed understood
**Down-regulated**	*LTA*	member of the tumor necrosis factor family, is a cytokine produced by lymphocytes; it has been described as inductor of apoptosis and as negative regulator of fibroblast proliferation
	*CASP8*	member of the cysteine-aspartic acid protease (caspase) family. This protein is involved in the programmed cell death induced by Fas and various apoptotic stimuli
	*CASP1*	member of the cysteine-aspartic acid protease (caspase) family
	*BCL2L11*	belongs to the BCL-2 protein family and act as an apoptotic activator
	*TP73*	a member of the p53 family of transcription factors involved in cellular responses to stress and development
	*HRK*	activator of apoptosis, HRK regulates apoptosis through interaction with death-repressor proteins Bcl2 and BCL-XL
	*CARD6*	microtubule-associated protein that has been shown to interact with receptor-interacting protein kinases. It plays pivotal role in signal transduction leading to apoptosis, NF-kB activation and inflammation

qRT-PCR confirmed the up-regulation of anti-apoptotic genes, such as *Bcl-xL, Bcl2* and *BIRC8* and the down-regulation of genes that have a central role in the execution-phase of cell apoptosis (*Casp1, Casp8 and LTA*) in TECs treated with cisplatin plus MVs in respect to TECs treated with cisplatin alone (Figure 5B and C).

To obtain *in vitro* evidence of *de novo* human protein expression in murine TECs by MV-mediated horizontal transfer of mRNA, we used as reporter genes *POLR2E* and *SUMO-1*. Human *POLR2E* mRNA was detected by RT-PCR after 1 hour of MV incubation with TECs (Figure 3). The primers used did not recognize murine mRNA as seen by negative RT-PCR in RNA extracted from control murine TECs, treated with cisplatin. *De novo* cytoplasmic and nuclear expression of human POLR2E and SUMO-1 proteins were detected in murine TECs after 24 hours incubation with cisplatin and MVs (Figure 3).

## Discussion

Several studies demonstrated that the administration of MSCs reverses AKI in different experimental models [2]–[7]. These beneficial effects were shown to be associated with the re-entry into cycle of renal tubular cells survived to injury [8]. The mechanisms have been mainly ascribed to a paracrine support of MSC to kidney repair. Consistently, Bi et al. [4] demonstrated that the administration of conditioned medium from MSCs may mimic the beneficial effects of the MSC administration, indicating that the tubular engraftment of the MSCs is not necessary. We recently reported that intravenously administration of MVs derived from human MSCs, has the same efficacy of MSCs on the functional and morphological recovery of glycerol-induced AKI in SCID mice [9].

Little is known at present on the biogenesis and the molecular composition of MVs produced by stem cells in different environmental conditions. It is suppose that the production of MVs is enhanced after appropriate stimulation. In the present study the serum starvation of MSCs was used as stimulus to enhance MV production and the effect of MVs was evaluated in a lethal model of AKI induced by cisplatin administration. Previous studies demonstrated that the administration of MSCs (intravenously or intraperitoneally) improves the survival in cisplatin induced AKI [2], [3] by a paracrine mechanism [4], [23]. We found that MSC-derived MVs significantly improved survival in cisplatin treated SCID mice. Two different regimens of MVs injection were used. The single administration of MVs ameliorated renal function and morphology and improved survival. However, at day 21 cisplatin treated mice given siMVs, showed chronic tubular injury and persistent increase in BUN and creatinine. When MVs were administered with multiple injections, the mortality further decreased and at day 21 survived mice showed normal histology and renal function. The mechanism of protection as judged by the significant decrease of Tunel positive cells in all survived mice was mainly ascribed to an anti-apoptotic effect of MVs. *In vitro* studies further supported this mechanism. Indeed, it has been shown that cisplatin induces death of TECs by a mechanism of apoptosis [24]. We found that incubation of cultured human TECs with MVs derived from MSCs significantly inhibited *in vitro* apoptosis induced by cisplatin. This effect was associated with the down-regulation of caspase-1 in tubular cells. Caspase-1 has been described as the main mediator of *in vitro* and *in vivo* cisplatin- [25] as well as ischemic-induced AKI [26]. Moreover, caspase-1 deficient mice are functionally and histologically protected against cisplatin-induced AKI [25]. For this reason, caspase-1 may be an important target for the development of inhibitors which might prevent cisplatin-induced AKI. In this study, we show that MVs could prevent *in vitro* cisplatin-induced tubular apoptosis by down-regulating caspase-1 mRNA. These data suggests that MVs could mediate, at least in part, the protective effect of MSCs in this model of renal injury. 

As previously reported a species-specificity of MVs was not observed as MVs derived from human MSCs were incorporated and transferred human transcripts in mice cells both *in vitro* and *in vivo* [9], [15], [20].

RNA inactivation in MVs reduced the *in vivo* effect on survival and functional and morphological recovery induced by MVs, suggesting a mechanism dependent on RNA delivery. Although MVs protect RNAs from physiological concentrations of RNase, the pre-treatment of MVs with high concentration of RNase can inactivate the RNAs [9], [10], [16], [20]. Bioanalyzer RNA profile of RNase-treated MVs showed a reduction of 18S and 28S ribosomal RNA residues and the quantitative RT-PCR showed significant reduction of the *ACT B, POLR2E* and *SUMO-1* mRNA content taken as reporter mRNA.

*In vitro* evidence for an effective horizontal transfer of mRNA was obtained by the presence of the human specific mRNA for *POLR2E* and by the *de novo* human protein expression of human POLR2E and SUMO-1 in MV-treated cisplatin TECs. This was confirmed *in vivo* where human POLR2 and SUMO-1 proteins were expressed by tubular cells of mice with cisplatin induced-AKI treated with MVs. We previously demonstrated that selected miRNAs enriched within human MVs are transferred to renal tubular epithelial cells of mice and are functional on their specific targets [18].

In conclusion, MVs released from bone marrow-derived MSCs were found to exert a pro-survival effect on renal cells *in vitro* and *in vivo*, suggesting that MVs may contribute to protection from AKI conferred by MSCs. Further studies are needed to investigate whether MVs may find a potential clinical application in human AKI.

## Materials and Methods

### Isolation and characterization of bone marrow MSCs

MSCs were obtained from Lonza (Basel, Switzerland), cultured and characterized as previously described [9], [10]. Briefly, the MSCs were cultured in the presence of Mesenchymal Stem Cells Basal Medium (MSCBM, Lonza). To expand the MSCs, the adherent monolayer was detached by trypsin treatment for 5 minutes at 37°C, after 15 days for the first passage and every 7 days for subsequent passages. Cells were seeded at a density of 10,000 cells/cm^2^ and used within the passage six. At each passage, cells were counted and analyzed for immunophenotype by cytofluorimetric analysis. The following antibodies, all phycoerythrin (PE) or fluorescein isothiocyanate (FITC) conjugated were used: anti-CD105, -CD146, -CD90 (Miltenyi Biotech, Bergisch Gladbach, Germany); -CD29, -CD44, -CD73, -CD34, -CD45, -CD80, -CD86, -CD166, HLA-I (Becton Dickinson Biosciences Pharmingen, San Jose, CA). Mouse IgG isotypic controls were from Dakocytomation (Copenhagen, Denmark).

All the cell preparations at different passages of culture expressed the typical MSC markers: CD105, CD73, CD44, CD90, CD166 and CD146. They also expressed HLA class I. MSC preparations did not express hematopoietic markers like CD45, CD14 and CD34. They also did not express the co-stimulatory molecules (CD80, CD86 and CD40).

The adipogenic, osteogenic and chondrogenic differentiation ability of MSCs was determined as previously described [9].

Human fibroblasts from dermas, used us control, were obtained from Lonza and maintained in DMEM (Sigma, St. Lousi, MO) with 10% FCS (Euroclone, Wetherby, UK) [9].

### Isolation and characterization of MVs

MVs were obtained from supernatants of MSCs and of fibroblasts, cultured overnight in RPMI deprived of Fetal Calf Serum (FCS) and supplemented with 0.5% of BSA (Sigma). The viability of cells incubated overnight without serum was 99% for MSCs and 85±4.3% for fibroblast as detected by trypan blue exclusion [9]. No apoptotic cells were detected by Tunel assay in MSCs and 2.8±1.3% apoptotic cell were detected for fibroblast. To obtain MVs, after centrifugation at 10,000 g for 20 minutes to remove debris, cell-free supernatants were centrifuged at 100,000 g (Beckman Coulter Optima L-90K ultracentrifuge) for one hour at 4°C, washed in serum-free medium 199 containing N-2-hydroxyethylpiperazine-N’-2-ethanesulfonic acid (HEPES) 25 mM (Sigma) and submitted to a second ultracentrifugation in the same conditions. The protein content of MVs was quantified by Bradford method (BioRad, Hercules, CA, USA). Endotoxin contamination of MVs was excluded by Limulus test according to the manufacturer’s instruction (Charles River Laboratories, Inc., Wilmington, MA, USA) and MVs were stored at -80°C. FACS analyses on isolated MVs were done as described [9], [10]. Briefly, by cytofluorimetric analyses MVs were detected mainly below the forward scatter signal corresponding to 1-µm beads. By Zetasizer Nano (Malvern Instruments, Malvern Worcestershire, United Kingdom), that is an instrument that permit to discriminate micro-particles inferior to 1 µm of diameter, the size of MVs ranged from 80 nm to 1 µm, with a mean value of 135 nm. Transmission and scanning electron microscopy performed on purified MVs showed their spheroid morphology and confirmed their size [9]. Cytofluorimetric analyses showed the presence of several adhesion molecules known to be expressed on MSC plasma membrane such as CD44, CD29, α4- and α5 integrins and CD73, but not α6-integrin [9]. In addition, MVs did not express HLA-class I at variance with the cells of origin or HLA-class II. The morphological analyses performed on MV suspension after staining with propidium iodide did not show the presence of apoptotic bodies.

We previously characterized the MVs content of mRNA, by microarray analysis [9], and of microRNA [18].

**Figure 6:**
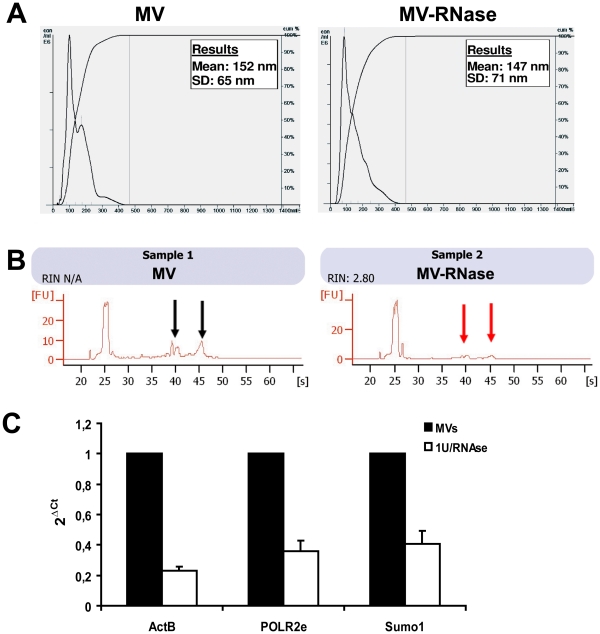
RNase treatment does not modify MV size, but reduces RNA content of MVs. A) Representative MV size analyses by direct measurement with NTA, showing no difference among MVs treated or not with RNase. B) Representative Bioanalyzer profile, showing the size distribution of total RNA extracted from MVs treated or not with RNAse. The first peak (left side of each panel) represents an internal standard. The two peaks in Sample 1 (black arrows) represent 18 S (left) and 28 S (right) ribosomal RNA, only partially detectable in MVs. The red arrows showed the reduction of 18 and 28 S fragment inside RNAse-treated MVs. C) Histogram showing the expression level of *SUMO-1*, *POLR2* and *Act B* transcripts in MVs treated or not with RNase, express as 2^-δCt^, as described in material and methods.

In selected experiments MVs were treated with 5U RNase (Ambion Inc., Austin, TX, USA) for 3 h at 37°C; the reaction was stopped by addition of 10 U/ml RNase inhibitor (Ambion) and MVs were washed by ultracentrifugation [9]. Treatment with RNase did not affect MVs morphology and surface protein expression, evaluated by FACS analyses, as previously reported [9]. For MVs size and morphology determination, nanoparticle tracking analysis (NTA) was performed using NanoSight LM10 instrument (NanoSight Ltd., Amesbuty, UK) equipped with the NTA 2.0 analytic software [27]. The analyses of MVs, treated or not with RNase, using NanoSight confirmed that RNase-treated MVs maintained the same physical characteristics of untreated MVs (Figure 6A).

Total RNA was isolated from MVs, treated or not with RNase, using the mirVana RNA isolation kit (Ambion) according to the manufacturer’s protocol. RNA integrity and structure and the efficacy of RNase treatment were evaluated by Agilent 2100 bioanalyzer (Agilent Technologies Inc., Santa Clara, CA), using the eukaryotic total RNA 6000 Pico Kit (Agilent Tech.). RNase treatment reduced 18 and 28S ribosomal RNA residues (Figure 6B).

Quantitative real time-PCR (qRT-PCR) was then performed to detect mRNA shuttle by MVs, such as *ACT B, POLR2E* and *SUMO-1* transcripts. The primers used for qRT-PCR are shown in Table 3. First-strand cDNA was produced from 100 ng of total RNA using High Capacity cDNA Reverse Transcription Kit (Applied Biosystems, Foster City, CA). Real-time PCR experiments were performed in 20 µl reaction mixture containing 5 ng of cDNA template, the sequence-specific oligonucleotide primers (purchased from MWG-Biotech AG, Ebersberg, Germany, www.mwg-biotech.com) and the Power SYBR® Green PCR Master Mix (Applied Biosystems). Negative cDNA controls (no cDNA) were cycled in parallel with each run. qRT-PCR was performed using a 48-well StepOne™ Real Time System (Applied Biosystems). As shown in figure 6C RNase treatment significantly reduced the *ACT B*, *POLR2E* and *SUMO-1* mRNA content in respect to untreated MVs. Fold change in mRNA expression was calculated based on cycle threshold (Ct) differences between treated and untreated MVs, loading the same quantity of RNA during the reverse transcription procedure.

**Table 3:** Primers used in qRT-PCR experiments.

Name	Official symbol	Sequences
**Polymerase (RNA) II (DNA directed)**	***POLR2E***	Forward 5'-GCTCTGGAAAATCCGCAAGA-3'Reverse 5'-TCCTCCAGGGTCTGGTCAAG-3'
**Senp2**	***SUMO-1***	Forward 5'-AAATAAGATCGACCAATGCAAGTG-3'Reverse 5'-TCACAGTCCAGGAGTGAAGTAATCA-3'
**Actin B**	***ACT B***	Forward 5'-TGA AGATCAAGATCATTGCTCCTC-3'Reverse 5'-CACATCTGCTGGAAGGTGGAC-3'
**Caspase 1**	***CASP1***	Forward 5′-CCGCAAGGTTCGATTTTCAT-3'Reverse 5′-TTTTAATGTCCTGGGAAGAGGTAGA-3'
**Lymphotoxin alpha (TNF superfamily, member 1)**	***LTA***	Forward 5′-CACCTCATTGGAGACCCCAG-3'Reverse 5′-TGGGAGTAGACGAAGTAGATGCC-3'
**Caspase 8**	***CASP8***	Forward 5'-TGATGACATGAACCTGCTGGAT-3'Reverse 5'-TGTCATTACCCCACACAACTCCT-3'
**Baculoviral IAP repeat-containing 8**	***BIRC8***	Forward 5′-ACCTGACCATTGAGGACCTGG-3'Reverse 5′-TGTAGTCGTGGCCCTGCTTC-3'
**B-cell CLL/lymphoma 2**	***Bcl2***	Forward 5′-GGAGGCTGGGATGCCTTT-3'Reverse 5′-GCCAAACTGAGCAGAGTCTTCA-3'
**BCL2-like 1**	***BCL2L1(BCL-XL)*** ** longer isoform**	Forward 5′-GGCTGGGATACTTTTGTGGAAC-3'Reverse 5′-ACAGTCATGCCCGTCAGGA-3'
**Glyceraldehyde-3-phosphate dehydrogenase**	***GAPDH***	Forward 5′-TGGAAGGACTCATGACCACAGT-3‘Reverse 5′- CATCACGCCACAGTTTCCC-3‘

### Murine model of acute kidney injury

Animal studies were conducted in accordance with the National Institute of Health Guide for the Care and Use of Laboratory Animals. The protocol was approved by the Committee on the Bioethics of the University of Torino (Permit Number: 1.3.10).

Models of AKI were performed in male SCID mice (7–8 weeks old) by subcutaneous injection of a single dose of 12 mg/kg of cisplatin (Sigma) dissolved in 0.9% saline solution. The dose was chosen on the basis of preliminary experiments using different doses of cisplatin (from 10 mg/kg to 20 mg/kg). SCID mice were kept in our institutional animal facility under well-controlled conditions of temperature (22±2°C), humidity (55±5%) and a 12 h/12 h light-dark cycle with access to food and water *ad libitum*.

Two regimens of MV administration were used (Figure 1):

single injection of MVs (siMVs): a single dose of 100 µg of MVs, or MVs pre-treated with RNase, was injected into the tail vein eight hours after cisplatin administration;
multiple injection of MVs (miMVs): a first intravenous (iv) injection of 100 µg of MVs was performed eight hours after cisplatin administration followed by iv injection of 50 µg of MVs at days 2, 6, 10, 14 and 18 after cisplatin administration.

Mice (8/group) were killed 4 days after MV administrations, and kidneys and samples for blood urea nitrogen (BUN) and creatinine determination were collected.

For survival studies, eight cisplatin treated mice were given with vehicle alone, eight cisplatin treated mice with siMVs pre-treated with RNase, fifteen cisplatin mice with siMVs and twelve cisplatin mice with miMVs. The animals were monitored for activity and physical conditions every day. Mice were followed for up to 21 days after treatments. Each animal was scored using an assessment form that evaluated each animal's health as described [28]. The animal were euthanized through CO2 inhalation.

### Renal function

Blood samples for measurement of BUN and plasma creatinine were collected 4, 14 and 21 days after cisplatin treatment. Serum creatinine was measured using a colorimetric microplate assay based on the Jaffe reaction (Quantichrom Creatinine Assay, BioAssay Systems, Hayward, CA, USA). BUN was measured by direct quantification of serum urea with a colorimetric assay kit according to the instruction protocol (Quantichrom Urea Assay, BioAssay Systems).

### Morphological studies

For renal histology 5 µm-thick paraffin kidney sections were routinely stained with hematoxylin and eosin (Merck, Darmstadt, Germany). Luminal hyaline casts and cell lose (denudation of tubular basement membrane) were assessed in non-overlapping fields (up to 28 for each section) using a 40× objective (high power filed, HPF). Number of casts and tubular profiles showing necrosis were recorded in a single-blind fashion [4], [9].

Apoptosis was measured by terminal transferase-mediated dUTP nick-end labeling (Tunel) assay (ApopTag Apoptosis Detection Kit; Millipore Inc., Billerica, MA, USA) according to the manufacturer protocol. Scoring Tunel-positive cells was carried out by counting the number of positive nuclei per field in 10 randomly chosen sections of kidney cortex using ×40 magnification.

Immunohistochemistry for the detection of proliferation of tubular cells was performed as previously described [4], [9]. Briefly, kidney sections were subjected to antigen retrieval, and slides were blocked and labelled with 1∶25 dilution of monoclonal anti-BrdU antibody (Dakocytomation) or 1∶400 of monoclonal anti-PCNA (Santa Cruz Biotechnology, Santa Cruz CA, USA). Immunoperoxidase staining was performed using 1∶300 dilution of anti-mouse HRP (Pierce, Rockford IL, USA). Scoring for BrdU- and PCNA -positive cells was carried out by counting the number of positive nuclei per field in 10 randomly chosen sections of kidney cortex using ×40 magnification. 

Confocal microscopy analysis was performed on frozen sections for the detection of specific human proteins POLR2E and SUMO-1, used as reporters, since their mRNAs are present in MVs derived from human MSCs, but not in murine tubular epithelial cells. Section were blocked and labelled with rabbit anti-human POLR2E (Abcam, Cambridge Science Park, UK) (1∶300 dilution) or rabbit anti-human SUMO-1 (Abcam) (1∶300 dilution). Omission of the primary antibodies or substitution with non immune rabbit IgG was used as controls. Alexa Fluor 488 anti-rabbit (Molecular Probes, Leiden, The Netherlands) was used as secondary antibody. Confocal microscopy analysis was performed using a Zeiss LSM 5 Pascal Model Confocal Microscope (Carl Zeiss International, Germany). Hoechst 33258 dye (Sigma) was added for nuclear staining.

### Apoptosis assays on human tubular epithelial cells

Primary cultures of human TEC were obtained from kidneys removed by surgical procedures and characterized as previously described [29], [30]. TECs were seeded at 4,000 cells/well into 96-well plates in DMEM (Sigma) in the presence of different doses of MVs derived from MSCs or from fibroblasts (10 or 30 µg/ml). Apoptosis was evaluated using the Tunel assay (ApopTag Apoptosis Detection Kit) 48 hours after the beginning of the experiments, as previously described [9]. We used, as apoptotic stimuli, serum deprivation, or stimulation with 5 µg/ml of cisplatinum in DMEM plus 3% fetal calf serum (FCS).

### Immunofluorescence for human protein expression on TECs

Indirect immunofluorescence was performed on TECs cultured on chamber slides (Nalgen Nunc International, Rochester, NY, USA) and stimulated for 1 day in the presence of 5 µg/ml of cisplatin (Sigma) and 50 µg of different preparations of MVs. The cells were fixed in 4% paraformaldehyde containing 2% sucrose and permeabilized with Hepes-Triton ×100 buffer (Sigma). The following antibodies were used: rabbit anti-human POLR2E (Abcam) and rabbit anti human SUMO-1 (Abcam). Omission of the primary antibodies and substitution with non immune rabbit IgG were used as controls. Alexa Fluor 488 anti-rabbit (Molecular Probes) was used as secondary antibody. Hoechst 33258 dye (Sigma) was added for nuclear staining.

### Reverse transcriptase PCR (RT-PCR)

Total RNA extracted from TECs, after incubation with 5 µg/ml of cisplatin and 250 µg of MVs for 1 hour, was submitted to RT-PCR [9] using the primer for human *POLR2E* reported in table 1. Bands of the expected size (90 pb) were detected in a 4% Agarose gel after electrophoresis. cDNA from a preparation of human bone marrow MSC was used as positive control.

### Apoptosis RT_2_ profile PCR arrays on tubular epithelial cells

TECs were seeded into T75 flasks at 80% of confluence and treated with 5 µg/ml of cisplatin (Sigma) in the presence or absence of MVs (30 µg/ml) in DMEM plus 3% FCS for 24 hours. Untreated tubular cells were used as control. Total RNA was then isolated from different cell preparations using the mirVana RNA isolation kit (Ambion) and cDNA was synthesized from 1 µg of RNA imput. The expression of 84 apoptosis- related genes was examined using the RT_2_ Profiler PCR array (SABioscience, Qiagen, Valencia, CA, USA) according to the manufacturer instructions using the Applied Biosystems 7900HT real-time PCR instrument. Data analysis was performed using the online SABioscience software and the expression levels of the mRNA of each gene in cisplatin and cisplatin plus MVs treated cells were normalized using the expression of *GAPDH, ACT B, B2M* and *RPL13A* as housekeeping genes and then compared with the data obtained from untreated cells. The results were confirmed by qRT-PCR performed using individual RNA samples from each group of cells by a 48-well StepOne™ Real Time System (Applied Biosystems) [31]. The primers used for qRT-PCR are shown in Table 3.

### Statistical analysis

Results are generally expressed as mean±standard deviation (SD). Statistical analysis was performed by using the Student *t*-tests (unpaired, 2-tailed), ANOVA with Newmann-Keuls’ or ANOVA with Dunnet’s multicomparison tests as appropriate. A *p* value of<0.05 was considered significant. 

For survival experiments, a log-rank test was conducted, and *p*<0.05 was deemed significant.
